# Anti-stress and neuronal cell differentiation induction effects of *Rosmarinus officinalis* L. essential oil

**DOI:** 10.1186/s12906-017-2060-1

**Published:** 2017-12-22

**Authors:** Myra O. Villareal, Ayumi Ikeya, Kazunori Sasaki, Abdelkarim Ben Arfa, Mohamed Neffati, Hiroko Isoda

**Affiliations:** 1 Faculty of Life and Environmental Sciences, University of Tsukuba, 1-1-1 Tennodai, Tsukuba City, Ibaraki, 305-8572 Japan; 2 Alliance for Research on North Africa (ARENA), University of Tsukuba, 1-1-1 Tennodai, Tsukuba City, Ibaraki, 305-8572 Japan; 3 Graduate School of Life and Environmental Sciences, University of Tsukuba, 1-1-1 Tennodai, Tsukuba City, Ibaraki 305-8572 Japan; 4 Interdisciplinary Research Center for Catalytic Chemistry, National Institute of Advanced Industrial Science and Technology (AIST), 1-1-1 Azuma, Tsukuba City, Ibaraki 305-8571 Japan; 5 Faculty of Pure and Applied Sciences, University of Tsukuba, 1-1-1 Tennodai, Tsukuba City, Ibaraki 305-8572 Japan; 6 Range Ecology Laboratory, Arid Land Institute, 4119 Medenine, Tunisia

**Keywords:** Catecholamines, Cell differentiation, Inhalation, Nerve growth factor, Rosemary essential oil

## Abstract

**Background:**

Mood disorder accounts for 13 % of global disease burden. And while therapeutic agents are available, usually orally administered, most have unwanted side effects, and thus making the inhalation of essential oils (EOs) an attractive alternative therapy. *Rosmarinus officinalis* EO (ROEO), Mediterranean ROEO reported to improve cognition, mood, and memory, the effect on stress of which has not yet been determined. Here, the anti-stress effect of ROEO on stress was evaluated in vivo and in vitro.

**Methods:**

Six-week-old male ICR mice were made to inhale ROEO and subjected to tail suspension test (TST). To determine the neuronal differentiation effect of ROEO in vitro, induction of ROEO-treated PC12 cells differentiation was observed. Intracellular acetylcholine and choline, as well as the *Gap43* gene expression levels were also determined.

**Results:**

Inhalation of ROEO significantly decreased the immobility time of ICR mice and serum corticosterone level, accompanied by increased brain dopamine level. Determination of the underlying mechanism in vitro revealed a PC12 differentiation-induction effect through the modulation of intracellular acetylcholine, choline, and *Gap43* gene expression levels. ROEO activates the stress response system through the NGF pathway and the hypothalamus-pituitary-adrenal axis, promoting dopamine production and secretion. The effect of ROEO may be attributed to its bioactive components, specifically to α-pinene, one of its major compounds that has anxiolytic property.

**Conclusions:**

The results of this study suggest that ROEO inhalation has therapeutic potential against stress-related psychiatric disorders.

## Background

Emotional disorders, including depression, cause a huge burden on health worldwide. Several theories to explain the pathogenesis of depression have been proposed but because of the complexity, identification of specific mechanism of depression has been difficult. However, evidences suggest that stress and depression are associated with atrophy and loss of neuronal cells, as well as reduction in the volume of key brain structures. Chronic exposure to stress, for example, induces neuronal inflammation, neuronal degeneration, and brain micro-damage [1]. Recently approved antidepressants, such as serotonin selective reuptake inhibitors (SSRIs) or anxiolytics that are mostly administrated orally, are found to exert better therapeutic efficacy than previous medications. However, the unwanted side effects of these drugs, such as the amnestic effect of benzodiazepine [2], have not yet been entirely eradicated and their molecular and therapeutic mechanisms are not fully understood [3]. To resolve concerns on limited efficacy of existing drugs, new antidepressant agents or alternative agents with novel mechanisms that are more effective and without or fewer side effects are sought.

Essential oils (EOs) have a long history of use as folk medicine and are still attractive as a therapeutic agent against oxidative stress [4], tumorigenesis [5], stress-related disorders including depression and anxiety [6]. Both anecdotal and empirical literature reports some major EOs, on the psychological or behavioral effects, however the effect of many EOs remained largely undiscovered. *Rosmarinus officinalis* is herb used for culinary and medicinal purposes that contains polyphenols such as rosmarinic acid, carnosic acid, and luteolin and other water-soluble phytochemicals that exert several effects on psychiatric diseases or neurological function including neuroprotective, cognitive properties, anti-depressive and anti-anxiety effects [7–9]. *R. officinalis* EO (ROEO) has been reported to exhibit anti-proliferative, antioxidant and antibacterial activities [10], as well as improvement of cognition, mood, and memory function in healthy adults [11] and improvement of locomotor activity in mice [12]. Oral intake of ROEO showed anti-depressant-like effect in mice [13] but EOs terpenoids and ketones often exhibit acute toxicity even in very low concentration [14]. Inhalation of EO as a sedative [15–17], anxiolytic [18–20] and anti-stress [6, 21] therapy ensures effectivity without unwanted side effects and has an advantage in terms of the absence of pathological consequences on organs and tissues [14]. The effectiveness and detailed mechanism of inhalation of ROEO on psychological and neurological function however, remains unknown.

Here, we evaluated the effect of ROEO inhalation on the molecular mechanism by which ROEO alleviates stress in vitro using PC12 cells and in vivo using ICR mice, a mice strain widely used for behavioral tests. Psychiatric functions and neurophysiological mechanism were determined in vivo using tail suspension test (TST), and measurement of the catecholamine and corticosterone level, while the effect on neuronal differentiation was determined using activity of acetylcholinesterase (AChE) and neuronal elongation as indicators, in vitro. We hypothesized that ROEO actively enhance the neuronal activity in mice by promoting cell differentiation.

## Methods

### Preparation of essential oil (EO) samples

The *Rosmarinus officinalis* L. leaves collected from Matmata, Tunisia that were used in the extraction of the *R. officinalis* essential oil (ROEO) used in this study and authenticated by Prof. Mohamed Neffati (Arid Land Institute, Tunisia). Voucher specimens of the Rosemary leaf samples (UT-ARENA-00323) used in this study were deposited in the Alliance for Research on North Africa, University of Tsukuba, Japan. ROEO was extracted from dried plant material (leaves) by hydrodistillation method, and kept in the dark at 4 °C until use. Pure EO of lavender (*Lavandula angustifolia*) (LAEO) and almond (*Prunus dulcis*) oil (AL) were purchased from Sigma (St Louis, MO, USA) and Wako Pure Chemical Industries, Ltd., (Osaka, Japan), respectively.

ROEO components were analyzed using Agilent 6890 N Network gas chromatographic (GC) system gas chromatograph equipped with a HPMS 5973 mass spectrometer with a HP-5MS fused silica column (30 m × 0.25 mm ID, film thickness 0.25 μm, Hewlett-Packard; carrier gas was helium adjusted to a linear velocity of 34 cm/s). The oven temperature program was the same as that used for the HP-5 column for GC-FID analysis: source temperature was 230 °C, quadrupole temperature was 150 °C, injector and detector was 250 °C and 280 °C respectively. Samples (0.1 μl) were injected with a split ratio of 1:100. The composition of the ROEO was identified by GCMS and the main components of the ROEO were identified to be camphor (20.42%), alpha-pinene (10.49%), beta-pinene (10.21%) (Table 1).

**Table 1:** GCMS analysis of the components of the *R. officinalis* essential oil (ROEO)

Compound	RT	Amount present ROEO (%)
Alpha-pinene	6.449	10.49
Beta-pinene	6.788	10.21
Camphene	7.360	3.13
O-cymene	8.515	3.56
Camphor	11.258	20.42
Borneol	11.644	5.57
Terpineol-4	11.835	2.25
Bornyl acetate	14.011	4.46
Alpha-eudesmol	20.662	2.38

### Animals

Six-week-old male ICR mice were used in this study (Charles River Laboratories Japan Inc., Yokohama, Kanagawa, Japan) and were housed individually in polycarbonate cage lined with paper bedding (Palsoft, Oriental Yeast Co., Ltd., Tokyo, Japan) and with a stainless steel wire cover. The mice were given access to water and food ad libitum under a 12/12 h light/dark cycle in a temperature- and humidity-controlled animal facility of the Gene research Center of the University of Tsukuba. Before the experiments, animals were allowed to acclimatize to laboratory condition for 1 week and were randomly assigned to experimental groups. Each mouse was sacrificed by cervical spine dislocation. Blood was collected and blood serum was collected by centrifugation at 2000×g for 10 min. Brain samples were collected and washed with PBS (−), then immediately immersed in liquid nitrogen and kept at −80 °C until use. The experiments performed in this study were approved by the Animal Care and Use Committee of the University of Tsukuba.

### EO inhalation method

EOs were dropped on the piece of cotton (Chiyoda, Osaka, Japan) placed on the upper part of the chamber before putting the mice inside the chamber (30 cm × 23 cm × 15 cm) [6] and allowed to inhale the EO for 30 min. Control group mice (*n* = 6) were placed in the chamber without the EOs. The volume of EOs that diffused in the chamber are as follows: 100 μl LAEO (*n* = 8), 100 μl AL (n = 8) and 50 μl ROEO (n = 8) or 100 μl ROEO (n = 8). AL was used as a negative control while LAEO was the positive control. The mice inhaled the EOs every day for 14 days. All mice have never been exposed to EO prior to this study.

### Tail suspension test (TST)

TST, a widely accepted method used for screening of antidepressants or anxiolytics in rodents, was performed following the method described by [22] Cavanagh et al. (2002). After EO inhalation, the mice were suspended for 6 mins by taping the tail to the hooks attached to the ceiling of the apparatus (30 × 15 × 50 cm; L × W × H) and their immobility time in the last 4 min was recorded, as described previously [8]. The immobility time measurement starts when the mice show no movement without postural distortion. TST was performed every other day for 2 weeks, the order of the treatment and testing was also done not in any particular order (random) until all the mice have been treated or tested.

### Serum corticosterone assay

Serum corticosterone (ng/ml) level was determined using AssayMax Corticosterone ELISA kit (AssayPro LLC, Saint Charles, MO, USA) following the manufacturer’s instructions. The serum was prepared as described previously [9]. Briefly, blood was centrifuged for 10 min at 2000×g to collect the serum samples which were stored in -80 °C prior to analysis. The corticosterone level was calculated based on the standard curve prepared using corticosterone standard reagent included in the kit.

### Dopamine (DOP), noradrenaline (NAD) and adrenaline (ADR) assay

Mice brains were collected and 100 mg sections of hippocampus and cerebral cortex were taken for analysis. To prepare the samples for analysis, 100 μl of distilled water was added to each sample, followed by homogenization for 1 min on ice. For the analysis of dopamine (DOP), noradrenaline (NAD) and adrenaline (ADR) levels, 3-CAT Research ELISA (LDN, Nordhorn, Niedersachsen, Germany) was used following the manufacturer’s instructions.

### Cell culture and sample preparation

PC12 cells (Riken, Tsukuba, Ibaraki, Japan) were maintained in Dulbecco’s Modified Eagle Medium (DMEM) (Sigma) supplemented with 5% heat-inactivated fetal bovine serum (FBS) (Bio West, USA), 10% heat-inactivated horse serum (HS) (Invitrogen, New Zealand) and 50 U/ml penicillin, 50 μg/ml streptomycin (Lonza Inc., Walkersville, MD, USA) and incubated at 37 °C in a humidified incubator with 5% CO_2_. The cells between passages 3 and 8 were used for the experiments. ROEO was sterilized by filtration (0.22 μm filter) and emulsified in growth medium, diluted with 10% volume of ethanol, and homogenized on ice prior to use. Nerve growth factor 7 s (NGF) (Sigma) was diluted in the growth medium at 50 ng/ml.

### Assessment of cell viability

Cell viability was assessed using 3-(4, 5-dimethylthiazol-2-yl)-2, 5- diphenyltetrazolium bromide (MTT) assay. PC12 cells were seeded at 1 × 10^5^ cells/ml into poly L-lysine-coated 96-well plates (Wako). After overnight incubation, cells were treated with or without ROEO at 5, 10, 50 and 100 μg/ml for 48 h. MTT assay was then performed by adding 10 μl of MTT (Dojindo, Japan) solution and incubating the cells further for 24 h at 37 °C in the dark., followed by addition of 100 μl of 10% SDS (Wako) and incubation for 24 h. The absorbance at 570 nm was determined using a multidetection microplate reader (Powerscan HT, Dainippon Pharmaceutical, Osaka, Japan). The viability of PC12 cells was calculated as percentage of control.

### Acetylcholinesterase (AChE) assay

Differentiating PC12 cells synthesize neurotransmitters that can be used as a marker of neuronal differentiation. PC12 cells were cultured as described above and treated without or with nerve growth factor (NGF), the positive control, or 10 μg/ml ROEO and incubated further for 48 h. The effect of ROEO on neuronal differentiation, using AChE assay, was performed as previously reported [23].

### Determination of intracellular choline (Ch) and acetylcholine (ACh) levels

The intracellular concentration of ACh and Ch in PC12 cells was measured using Ultimate 3000 Dionex HPLC coupled with electro-chemical detector (ECD). PC12 cells were seeded at 1 × 10^5^ cells/ml into 10-cm dish and incubated overnight. Then, cells were treated as described above and washed with PBS(−) twice and collected by trypsination. Cells were homogenized in 15% formic acid in acetone and centrifuged at 12,000×g, 4 °C for 15 min. The supernatant was evaporated using a vacuum centrifuge (SCRUM Inc., Japan) and the precipitate dissolved in 100 μl of milliQ water and filtered and analyzed for intracellular level of ACh and Ch using AFpack ACH-494 column (4.6 mm ID ×10 mm L) (Shodex, Japan) was used. The amount of ACh and Ch was measured as described previously [9].

### Measurement of growth associated factor 43 (*Gap43*) gene expression

To reveal the effect of ROEO on neuronal differentiation at the transcription level, the expression of *Gap43* gene was quantified using real-time PCR. PC12 cells were seeded and treated as described above. After treatment, the cells were washed with PBS(−) twice and detached by gently scraping the cells. RNA was extracted using ISOGEN (NIPPON GENE CO. Ltd) following the manufacture’s instruments. Reverse transcription was performed using Superscript III reverse transcriptase kit (Invitrogen, Carlsbad, CA, USA) and amplified using 2720 Thermal Cycler (Applied Biosystems, USA). Real-time PCR amplification reactions were performed using an AB7500 Fast real-time system (Applied Biosystems). Using the following cycling conditions: 2 min at 50 °C, 10 min at 95 °C, and 40 cycles of 15 s at 95 °C/1 min at 60 °C. Specific for rat growth associated protein 43 (*Gap43*) (Rn01474579_m1) and *Gapdh* (Rn01775763_g1) were purchased from Applied Biosystems.

### Effect of ROEO on differentiated neuronal cells

To determine the effect of ROEO on neuronal cells, PC12 cells were treated with 50 ng/ml NGF (NGF) to induce the cells to differentiate while untreated cells served as the control. The cells were then incubated further for 24 h, followed by treatment with (NGF + ROEO) or without 10 μg/ml ROEO (ROEO) for 48 h. The level of AChE, which indicates the induction activity of neuronal differentiation of PC12 cells, was then quantified using the AChE assay. AChE activity was assessed using the protocol described above.

### Statistical analysis

All the results were expressed as mean ± standard deviation. Statistical analysis was performed using Student’s *t*-test. Comparisons between groups were carried out using one way analysis of variance (ANOVA) (One-Way ANOVA, Inferential Statistics Computational Suite version 10.4.0) followed by a pairwise comparison using the JMP Statistical Discovery software from SAS version 13.2.0. *P* ≤ 0.05 was considered to be statistically significant.

## Results

### ROEO alleviated TST-induced stress in vivo

**Fig. 1:**
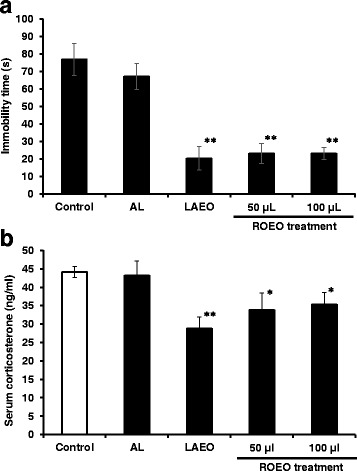
Effect of *Rosmarinus officinalis* essential oil (ROEO) on mice immobility time in tail suspension test (TST), and serum corticosterone level. **a** Effect of ROEO on the immobility time of mice in tail suspension test (TST). Mice were made to inhale the EOs for 30 min daily for 2 weeks. The volume of EO for each experiment was 100 μl for almond oil (AL) and lavender EO (LAEO), and 50 or 100 μl for ROEO. During the two-week inhalation of EO, TST was performed every other day measuring the immobility time at the last 4 min of the six-min TST session. Data represent the average result of the TST performed on the last day of the experiment. Each result represents the mean ± .SD (*n* = 5). *P≤0.05, **P≤0.01 vs. control. **b** Effect of ROEO on mice serum corticosterone level. At the end of the two-week EO inhalation, mice were sacrificed and serum was collected. Serum corticosterone level was quantified using an ELISA kit (AssayMax Corticosterone ELISA Kit, AssayPro LLC, Saint Charles, MO, USA) as described in Materials and Methods. Data represent the mean of ±.SD (*n* = 3). *P≤0.05, **P≤0.01 vs. control

To assess the effect of ROEO inhalation on mice behavior, specifically on stress, TST was performed. TST is a well-accepted method for measuring stress in mice as well as their reaction to anti-depressants, with the immobility time considered as the behavioral index of animal’s ability for coping with stressful stimuli [24]. As shown in Fig. 1a, ROEO treatment at 50 μl/day and 100 μl/day decreased the immobility time to 20.13 s and 8.92 s, respectively, in comparison with the control (76.17 s). The average immobility time of the almond oil (AL) group was not significantly different compared to the control (61.75 s) while *L. angustifolia* EO (LAEO) decreased it to 17.36 s. Pairwise comparison analysis results revealed that LAEO, the positive control, was not was not significantly different to 50 and 100 μL ROEO (*P* = 0.59 and 0.53, respectively). Analysis of variance results showed significant differences in the immobility time between treatment groups (*P* = 0.0076). The body weight and the overall health condition of the animals and the amount of feeds consumed were measured throughout the duration of the study and no significant difference among all groups was recorded (data not shown).

### ROEO modulated the activity of HPA axis and brain catecholamine level in vivo

With the observed decrease in immobility time, we then wanted to verify if this was a result of any effect of ROEO on the HPA axis activity. Chronic elevated serum corticosterone level is an indication of hyperactivity of the HPA axis, which may lead to depressive-like behaviors, which increases immobility time in TST [24]. Corticosterone is a hormone associated with stress and is secreted when the hypothalamus-pituitary-adrenal (HPA) axis is activated [25]. The corticosterone levels of mice groups treated with 50 μl/day or 100 μl/day ROEO were 33.8 ng/ml and 35.3 ng/ml, respectively (Fig. 1b). The effect was not significant for the AL mice group (43.1 ng/ml) compared with the control group (44. 2 ng/ml). For LAEO group, the corticosterone level was 31.83 ng/ml. Mice that inhaled 50 μl/day and 100 μl/day ROEO and LAEO had significantly lower corticosterone level compared to the AL group. ROEO at 50 μl/day treatment showed lower corticosterone level compared to LAEO and was the most effective among these groups. The level of dopamine (DOP), noradrenaline (NAD) and adrenaline (ADR) in the mice brain were also evaluated and presented in Table 2.

**Table 2:** Catecholamine, dopamine (DOP), noradrenaline (NAD) and adrenaline (ADR) levels in mice brain (ng/100 mg brain tissue)^1^

Group	DOP^a^	NAD^b^	ADR^b^
Control	333.86 ± 12.32	44.31 ± 2.73	0.33 ± 0.057
AL	443.1 ± 5.65	48.81 ± 2.64	0.31 ± 0.051
LAEO	792.03 ± 14.49 *	55.29 ± 3.18 *	0.44 ± 0.042
ROEO 50	653.56 ± 8.22 *	50.74 ± 4.86	0.35 ± 0.071
ROEO 100	765.52 ± 12.92 *	46.64 ± 4.51	0.34 ± 0.067

^1^Data represent the mean ± SD. *P ≤ 0.05, **P ≤ 0.01 vs. control; ^a^ *P* ≤ 0.01 significant differences between groups, ^b^not significant differences between groups (ANOVA)

ROEO at 50 μl/day and 100 μl/day had significantly improved DOP level compared to the control (333.86 ng/100 mg). AL did not have significant effect on the levels of the neurotransmitters. Like ROEO, LAEO also improved the DOP level. Compared with LAEO (792.03 ng/g), both ROEO treatment groups showed lower DOP concentration (653.56 ng/100 mg and 765.52 ng/100 mg for 50 μl/day and 100 μl/day, respectively). With regards to the NAD levels, no significant effect was observed in ROEO (507.36 ng/100 mg and 46.64 ng/100 mg in 50 μl/day and 100 μl/day, respectively) group but a significant improvement was observed in LAEO group (55.29 ng/100 mg) compared with control group (44.31 ng/100 mg). For ADR, ROEO group did not show significant change (0.35 ng/100 mg and 0.34 ng/100 mg, in 50 μl/day and 100 μl/day ROEO, respectively), as with LAEO group (0.44 ng/100 mg LAEO) group compared to the control group (0.33 ng/100 mg). Both ROEO treatment groups showed lower level of ADR compared with LAEO. Inhalation of ROEO (50 μl/day and 100 μl/day) showed significant effects on serum brain DOP level. ANOVA results showed significant differences in the DOP levels between groups (Table 2).

### ROEO promoted acetylcholinesterase (AChE) activity and upregulated *Gap43* mRNA expression in PC12 cells

Observed improved corticosterone and dopamine levels, which could be due to neuronal differentiation, was observed following ROEO inhalation. To verify if ROEO induced neuronal differentiation, acetylcholinesterase (AChE) activity and the expression of *Gap43* mRNA expression in PC12 cells were determined. First, the non-cytotoxic concentration of ROEO was determined by performing MTT assay. PC12 cells were treated with or without ROEO (0, 5, 10, 50, and 100 μg/ml) for 48 h. MTT assay results showed that ROEO was not cytotoxic at low concentrations (5 and 10 μg/ml) but was slightly cytotoxic at 50 and 100 μg/ml, decreasing the cell viability to 97% and 81.41% for 50 and 100 μg/ml ROEO, respectively.

**Fig. 2:**
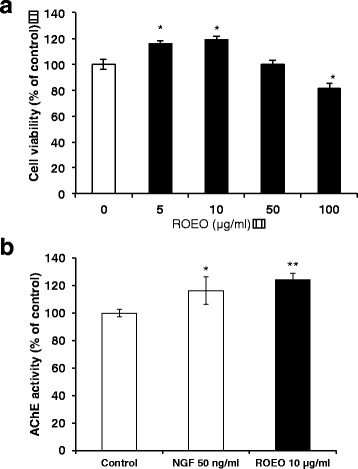
Effect of *Rosmarinus officinalis* essential oil (ROEO) on PC12 cell proliferation and acetylcholinesterase activity. **a** Effect of ROEO on PC12 cells viability. PC12 cells were seeded onto 96-well plates at a density of 1×10^5^ cells/ml. After overnight incubation, cells were treated with 0, 5, 10, 50 or 100 μg/ml ROEO for 48 h and the cell viability was evaluated using MTT assay. Data are presented as a percentage of the control and represent the mean of three independent trials ±.SD *P≤0.05 vs. control. **b** Effect of ROEO on the acetylcholinesterase (AChE) activity in PC12 cells. PC12 cells were seeded onto 96-well plates at a density of 1×10^5^ cells/ml. After overnight incubation, cells were treated with 50 ng/ml of nerve growth factor (NGF) or 10 μg/ml of ROEO for 48 h. The control indicates cells incubated for 48 h without NGF or ROEO treatment. AChE activity was expressed in percentage of control. Data represent the mean of three independent trials ±.SD *P≤0.05, **P<≤0.01 vs. control

The synthesis and secretion of neurotransmitters are significant events associated with neuronal differentiation. In PC12 cells, the secretion of acetylcholine (ACh) is induced following induction of PC12 cell differentiation. Treatment with μg/ml ROEO increased the AChE activity in PC12 (124.16%) compared both the control (100%) and 50 ng/ml NGF (116.36%) (Fig. 2b). Since ACh is produced from ingested choline (Ch) and from recycled choline in the synapse, we then determined the intracellular concentration of ACh and its substrate Ch using HPLC-ECD. As presented in Table 3, ROEO increased the intracellular ACh and Ch levels to 11.88 ng/mg and 2645.49 ng/mg, respectively. As expected, NGF increased the intracellular ACh (11.99 ng/mg) and Ch levels (2660.6 ng/mg) compared to the control. The control had ACh and Ch levels of 9.51 ng/mg and 2156.21 ng/mg, respectively.

**Table 3:** Intracellular acetylcholine (ACh) and choline (Ch) level in PC12 cells (ng/mg) ^a^

Group	Control	NGF (50 ng/ml)	ROEO (10 μg/ml)
Total choline (Ch)	2156.21 ± 237.73	2660.6 ± 178.16 *	2645.49 ± 244.18*
Acetylcholine (ACh)	9.51 ± 1.13	11.99 ± 2.78*	11.88 ± 2.26*

^a^Data represent the mean ± .SD. *P ≤ 0.05, **P ≤ 0.01 vs. control

**Fig. 3:**
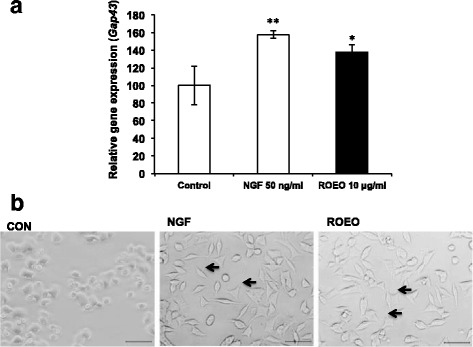
Effect of *Rosmarinus officinalis* essential oil (ROEO) on the PC12 cells neuronal outgrowth elongation.PC12 cells were seeded onto 96-well plates at a density of 1×10^5^ cells/ml. After overnight incubation, PC12 cells were treated with 50 ng/ml of nerve growth factor (NGF) or 10 μg/ml of ROEO for 48 h. Control cells were incubated for 48 h without NGF or ROEO treatment. **a** Expression of growth associated protein 43 (*Gap43*) mRNA quantified using real-time PCR and normalized to *Gapdh* mRNA expression. Data represent the mean of three independent trials ±.SD *P≤0.05, **P≤0.01 vs. control. **b** Morphology of PC12 cells treated without (Control) or with 50 ng/ml NGF (NGF) or 10 μg/ml of ROEO (ROEO). The photographs were taken 48 h after sample treatment and arrows point to cells with dendritic morphology. Photographs of the cells at 200× magnification were taken using a phase contrast microscope equipped with DFC 290HD camera (Leica Microsystem). The scale-bar represents 50 μm

**Fig. 4:**
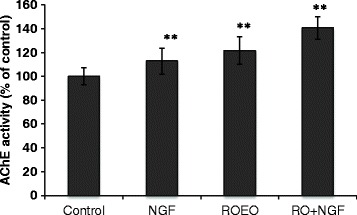
Effect of *Rosmarinus officinalis* essential oil (ROEO) on acetylcholinesterase (AChE) activity in differentiated PC12 cells. PC12 cells were seeded onto 96-well plates at a density of 1×10^5^ cells/ml and were pre-treated with 50 ng/ml nerve growth factor (NGF) for 24 h before treatment with 10 μg/ml of ROEO for 48 h without (ROEO) or with 10 ng/ml NGF (RO + NGF). AChE activity was expressed as percentage of control. Data are presented as a percentage of the control and represent the mean of three independent trials ±.SD *P≤0.05, **P≤0.01 vs. control

To further investigate the effects of ROEO on neuronal differentiation, the gene expression level of growth-associated protein 43 (*Gap43*) was determined. Quantitative real-time PCR results showed that NGF treatment significantly increased the *Gap43* expression by 38% by ROEO and by 57.37% compared to the control (Fig. 3a). ROEO treatment (10 μg/ml) and NGF treatment (50 ng/ml) induced PC12 cells to undergo neuronal outgrowth elongation as shown in Fig. 3b. And to evaluate the effect of ROEO on PC12 cells differentiation in vitro, cells NGF-differentiated cells were treated with ROEO and its effect on AChE expression was evaluated. AChE assay results showed that compared to the control (untreated cells), differentiated cells (+NGF) treated with 10 μg/ml ROEO significantly increased the AChE activity by 40.7% while treatment with NGF alone or ROEO treatment increased the AChE activity by only 12.9% and 21.5%, respectively (Fig. 4).

## Discussion

The demand for therapeutics for the cure and prevention of mental and neurological diseases has increased recently mainly due to the fact that most orally administrated drugs have adverse side effects. One alternative therapy that is gaining a lot of popularity is inhalation of essential oils (EOs) and fragrant compounds, the reports for which indicate their potential therapeutic effects on behavior, and on the serum corticosteroid and brain neurotransmitter levels [6, 19, 26, 27]. In the present study, the effect of ROEO on stress response system, including HPA axis and brain neurotransmitters, was evaluated. *R. officinalis* L. has been reported to activate cholinergic activity, as well as neuroprotective and anti-depressive effects [8, 23]. TST results revealed that ROEO could significantly decrease the mice immobility time by at least 50 s compared to the control mice (Fig. 1a). The same effect was observed in mice exposed to one of the positive controls, LAEO (17.36 s), and this effect was attributed to (−)-linalool, one of its active components that can decrease mice immobility time in TST [28]. TST and forced swimming test (FST) are well-known behavioral tests for assessing anti-depressant activity. TST was chosen to evaluate the effect of ROEO because of its several advantages compared to the FST. In FST the difference in the swimming ability of each animal can influence the results, and hypothermia may be also be induced [29]. Using TST, various antidepressant or anxiolytic medications have been demonstrated to decrease the immobility and promoted the escape-related behavior [24, 29]. The decreased immobility time observed in ROEO and LAEO inhalation suggests that they also have anti-stress effect.

The HPA axis is a key response system against stressors and plays an important role in the pathophysiology of stress-related mental disorders. Chronic stress results from high glucocorticoids level with the hyperactivity of HPA axis triggering several physiological adaptive feedback regulation mechanism [1, 30]. ROEO lowered the serum corticosterone level comparable to LAEO (Fig. 1b) and this result suggests that ROEO suppressed the activity of HPA axis increased by TST-induced stress.

ROEO regulated the brain catecholamine level, significantly increasing only the DOP level whereas LAEO increased the DOP, NAD, and ADR levels (Table 2), consistent with previous reports on LAEO [31]. The activity of sympathetic neuron is essential for coping with stress and is part of the stress response system. Studies have established the relationship between the amount of catecholamine in the brain and psychiatric diseases, a decrease in DOP level, specifically, is observed in individuals suffering from depression [32]. The brain catecholamine levels suggest that the anti-stress effect of ROEO appears to be different from that of LAEO, indicating a possible difference in the mechanism involved for these oils. Significant enhancement of memory was observed in ROEO-administered mice but not in LAEO group. Both essential oils, however, have been observed to have significant improvement on cognitive performance and mood in 144 healthy volunteers [11]. However, the effect of LAEO may be more similar to benzodiazepines in that it enhances the effects of gamma-aminobutyric acid in the amygdala region [22].

EOs may be administrated orally or as intraperitoneal injection, but inhalation is preferred with respect to safety and benefits. Fragrant compounds reach the central nervous system (CNS) through the olfactory and respiratory systems, introducing the drug by axonal transport or activity of perineural channels [33]. Orally administrated drugs usually have considerable acute oral toxicity [34].

Modulation of function of neuronal cells, like neurotransmission and synaptic plasticity, is directly correlated with the regulation of stress response systems, thereby preventing stress-related neurological diseases. Corticosterone hypersecretion, however, suppress the production of neurotrophic factors NGF and brain-derived neurotrophic factor (BDNF) [35]. NGF and BDNF are known to induce neuronal differentiation but cannot cross the blood brain barrier (BBB) easily, and thus, are metabolized by peptidases when administered peripherally [36]. However, when inhaled, some of the compounds present in EOs can reach the CNS, which suggests the possibility of inhalation of EOs as a better alternative therapy for neurological diseases [37–39]. Some of the active components of major EOs have been reported to pass the BBB, and this includes α-pinene that is present in ROEO [40]. Inhaled α-pinene has been shown to have anxiolytic effect in mice [41], and as one of the effective compounds in ROEO, α-pinene contributed to the observed effects of inhaled ROEO, but for the other compounds, synergistic effect of various compounds in the EO might have contributed to the effect [10] (Bakkali et al., 2008).

PC12 cells are known to differentiate into autonomic neuron-like cells, gaining function for neurotransmitter production, and elongation of neurite outgrowth when treated with NGF or NGF-like substances [42]. In this study, PC12 was used to evaluate the effect of the ROEO on neuronal differentiation. ROEO significantly increased both ACh and Ch levels (Table 3). Secreted ACh was observed to be higher in differentiated PC12 cells following ROEO treatment suggesting that the enzymatic activity of AChE was due to ROEO treatment (Fig. 2b). It can also be noted that in undifferentiated PC12 Ach was increased by ROEO, even without NGF to induce differentiation, providing further evidence of its differentiation induction effect that was also shown in Fig. 3b. In the ACh synthesis pathway, the uptake of Ch, regulated by the activity of choline acetyl transferase (ChAT), is the rate-limiting step [43]. AChE activity has been demonstrated to be affected in animal model of stress, so that an increase in the intracellular Ch and ACh level, following activation of AChE, the observed enhanced ACh synthesis and secretion was caused by the inhaled ROEO (Fig. 2b).

Growth associated protein 43 (*Gap43*) is a marker of neuronal outgrowth elongation as its expression is associated with neuronal differentiation, neuroplasticity and neuronal outgrowth elongation [44, 45]. ROEO upregulated *Gap43* expression and thus, provided additional evidence for its ability to induce neuronal differentiation (Fig. 4). Moreover, AChE activity in differentiated PC12 cells was significantly increased by ROEO treatment, suggesting its ability to promote cholinergic function following neuronal differentiation. ROEO is likely to exert an effect similar to NGF and it can be assumed that the mechanism of the effect of ROEO on neuronal cells might be related to NGF-mediated signaling pathways such as through ERK1/2, PI3K/AKT, and protein kinase C (PKC) which activity is reported to correlate with the survival of existing neuronal cells and neuronal cell differentiation [42]. Although the results have shown significant improvement in the mice’s stress levels following inhalation of ROEO, the procedure for the introduction of the EOs can be made more efficient by using a face mask-like instrument to ensure that the mice will be introduced to more EOs.

## Conclusions

The results of this study have shown that inhalation of ROEO alleviates stress by decreasing serum corticosterone level and increasing brain DOP level in vivo, suggesting that ROEO can regulate the activities of the HPA axis and sympathetic nerve system. Furthermore, in vitro, ROEO modulates brain neurotransmitter activity, exerts neurophysiological effect related with the activity of ACh synthesis and secretion, and induce neuronal differentiation. The increase in *Gap43* mRNA expression, in addition to elevation in the stress response and neuronal plasticity, further suggests the role of the HPA axis in ROEO’s anti-stress effect. A clinical trial may be useful in assessing the validity of the results in clinical depression. The results presented here suggest that ROEO has a potential for use as a safe alternative treatment for stress-related mood disorders.

## Abbreviations

ACh: Acetylcholine
AChE: Acetylcholinesterase
ACTH: Adrenocorticotrophic hormone
ADR: Adrenalin
AL: Almond
BDNF: Brain-derived neurotrophic factor
Ch: Choline
CNS: Central nerve system
DMEM: Dulbecco’s modified eagle medium
DOP: Dopamine
ECD: Electro chemical detector
EO: Essential oil
FBS: Fatal bovine serum
*Gap43*: *Growth associated protein 43*
*GAPDH*: Glyceraldehyde-3-phosphate dehydrogenase
HPA: Hypothalamus-pituitary-adrenal
HS: Horse serum
LA: *Lavandula angustifolia*
MTT: 3-(4,5-dimethylthiazol-2-yl)-2,5- diphenyltetrazolium bromide
NAD: Noradrenaline
NGF: Nerve growth factor
PC12: Rat pheochromocytoma
RO: *Rosmarinus officinalis* L.
SSRIs: Serotonin selective reuptake inhibitors
TST: Tail suspension test

## References

[1] Chovatiya R, Medzhitov R (2014). Stress, inflammation, and defense of homeostasis. Mol Cel.

[2] Verwey B, Muntendam A, Ensing K, Essink G, Pasker-de Jong PC, Willekens FL, Zitman FG (2005). Clinically relevant anterograde amnesia and its relationship with blood levels of benzodiazepines in suicide attempters who took an overdose. Prog Neuropsychopharmacol Biol Psych.

[3] Khawam EA, Laurencic G, Malone DA (2006). Side effects of antidepressants: an overview. Curr Drug Therapy.

[4] Raskovic A, Milanovic I, Pavlovic N, Cebovic T, Vukmirovic S, Mikov M (2014). Antioxidant activity of rosemary (*Rosmarinus officinalis* L.) essential oil and its hepatoprotective potential. BMC Complement Alt Med.

[5] Jeong JB, Choi J, Lou Z, Jiang X, Lee SH (2013). Patchouli alcohol, an essential oil of Pogostemon Cablin, exhibits anti-tumorigenic activity in human colorectal cancer cells. Int Immunopharmacol.

[6] Komiya M, Takeuchi T, Harada E (2006). Lemon oil vapor causes an anti-stress effect via modulating the 5-HT and DA activities in mice. Behav Brain Res.

[7] Akrout A, Mighri H, Krid M, Thabet F, Turki H, El-Jani H, Neffati M (2012). Chemical composition and antioxidant activity of aqueous extracts of some wild medicinal plants in southern Tunisia. International J Life Sci Med Sci.

[8] Kondo S, Omri AL, Han J, Isoda H (2015). Antidepressant-like effects of rosmarinic acid through mitogen-activated protein kinase phosphatase-1 and brain-derived neurotrophic factor modulation. J Funct Foods.

[9] Sasaki K, Omri A, Kondo S, Han J, Isoda H (2013). *Rosmarinus officinalis* polyphenols produce anti-depressant like effect through monoaminergic and cholinergic functions modulation. Behav Brain Res.

[10] Bakkali F, Averbeck S, Averbeck D, Idaomar M (2008). Biological effects of essential oils – a review. Food Chem Toxicol.

[11] Moss M, Cook J, Wesnes K, Duckett P (2003). Aromas of rosemary and lavender essential oils differentially affect cognition and mood in healthy adults. Int J Neurosci.

[12] Perry N, Perry M (2006). Aromatherapy in the management of psychiatric disorders. CNS Drugs.

[13] Machado DG, Cunha MP, Neis VB, Balen GO, Colla A, Bettio LEB, Olivera BA, Panzini FL, Dalmarco JB, Simionatto EL, Pizzolatti MG, Rodrigues AL (2013). Antidepressant-like effects of fractions, essential oil, carnosol and betulinic acid isolated from *Rosmarinus officinalis* L. Food Chem.

[14] Buchbauer G, Jirovetz L, Jäger W, Dietrich H, Plank C, Karamat E. Aromatherapy: evidence for sedative effects of the essential oil of lavender after inhalation. *Z* Naturfors. 1991;46c:1067–72. 10.1515/znc-1991-11-1223 1817516

[15] Carvalho-Freitas MIR, Costa M (2002). Anxiolytic and sedative effects of extracts and essential oil from *Citrus aurantium* L. Biol Pharma Bull.

[16] Ito K, Akahoshi Y, Ito M, Kaneko S. Sedative effects of inhaled essential oil components of traditional fragrance *Pogostemon cablin* leaves and their structure–activity relationships. J Tradit Complement Med. 2015; 10.1016/j.jtcme.2015.01.004. 10.1016/j.jtcme.2015.01.004 PMC4833466 27114936

[17] Wei T, Sun J, Han L, Chen K, Wang Z, Ji H (2015). Effects of the ornidazole enantiomers on the central nervous system: involvement of the GABAA receptor. Chemico-Biol Int.

[18] Galdino PM, Nascimento MVM, Florentino IF, Campos Lino R, Fajemiroye JO, Abdallah Chaibub B, de Paula JR, de Lima TCM, Costa EA (2012). The anxiolytic-like effect of an essential oil derived from a. St. Hil. Leaves and its major component, β-caryophyllene, in male mice. Progress Neuro-Psychopharmacol Biol Psych.

[19] Shaw D, Annett JM, Doherty B, Leslie JC (2007). Anxiolytic effects of lavender oil inhalation on open-field behaviour in rats. Phytomed..

[20] Silenieks LB, Koch E, Higgins GA (2013). Silexan, an essential oil from flowers of *Lavandula angustifolia*, is not recognized as benzodiazepine-like in rats trained to discriminate a diazepam cue. Phytomed.

[21] Takahashi M, Yoshino A, Yamanaka A, Asanuma C, Satou T, Hayashi S, Masuo Y, Sadamoto K, Koike K (2012). Effects of inhaled lavender essential oil on stress-loaded animals: changes in anxiety-related behavior and expression levels of selected mRNAs and proteins. Nat Prod Comm.

[22] Cavanagh HMA, Wilkinson JM (2002). Biological activities of lavender essential oil. Phytother Res.

[23] Omri AL, Han J, Yamada P, Kawada K, Abdrabbah MB, Isoda H (2010). *Rosmarinus officinalis* polyphenols activate cholinergic activities in PC12 cells through phosphorylation of ERK1/2. J Ethnopharmacol.

[24] Cryan JF, Mombereau C, Vassout A (2005). The tail suspension test as a model for assessing antidepressant activity: review of pharmacological and genetic studies in mice. Neurosci Biobehav Rev.

[25] De Kloet ER, Karst HM, Joëls M (2008). Corticosteroid hormones in the central stress response: quick-and-slow. Front Neuroendocrinol.

[26] Braden R, Reichow S, Halm MA (2009). The use of the essential oil lavandin to reduce preoperative anxiety in surgical patients. J Peri Anesthesia Nursing.

[27] Hao CW, Lai WS, Ho CT, Sheen LY (2013). Antidepressant-like effect of lemon essential oil is through a modulation in the levels of norepinephrine, dopamine, and serotonin in mice: use of the tail suspension test. J Funct Foods.

[28] Coelho V, Mazzardo-Martins L, Martins DF, Santos AR, Da Silvia Brum LF, Picada JN, Pereira P (2013). Neurobehavioral and genotoxic evaluation of (−)-linalool in mice. J Nat Med.

[29] Steru L, Chermat R, Thierry B, Simon P (1985). The tail suspension test: a new method for screening antidepressants in mice. Psychopharmacol.

[30] Du J, McEwen B, Manji HK (2009). Glucocorticoid receptors modulate mitochondrial function. Communic Integ Biol.

[31] Yamada K, Mimaki Y, Sashida Y (2005). Effects of inhaling the vapor of *Lavandula burnatii* super-derived essential oil and linalool on plasma adrenocorticotropic hormone (ACTH), catecholamine and gonadotropin levels in experimental menopausal female rats. Biol Pharm Bull.

[32] Hasler G, Fromm S, Carlson PJ, Luckenbaugh DA, Waldeck T, Geraci M, Roiser JP, Neumeister A, Meyers N, Charney DS, Drevets WC (2008). Neural response to catecholamine depletion in unmedicated subjects with major depressive disorder in remission and healthy subjects. Arch Gen Psychiat.

[33] Nan Lv, Zhu JL, Jing Zhang H, Tzeng CM. Aromatherapy and the Central Nerve System (CNS): Therapeutic Mechanism and its Associated Genes. Current Drug Targets. 2013;14(8):872–9. 10.2174/1389450111314080007 23531112

[34] Thibaut R, Porte C (2008). Effects of fibrates, anti-inflammatory drugs and antidepressants in the fish hepatoma cell line PLHC-1: Cytotoxicity and interactions with cytochrome P450 1A. Toxicology.

[35] Lanfumey L, Mongeau R, Cohen-Salmon C, Hamon M (2008). Corticosteroid–serotonin interactions in the neurobiological mechanisms of stress-related disorders. Neurosci Biobehav Rev.

[36] Hur JY, Lee P, Kim AJ, Kim H, Kim SY (2004). Induction of nerve growth factor by butanol fraction of *Liriope platyphylla* in C6 and primary astrocyte cells. Biol Pharm Bull.

[37] Kim KY, Seo HJ, Min SS, Park M, Seol GH (2014). The effect of 1,8-cineole inhalation on preoperative anxiety: a randomized clinical trial. Evidence-Based Complement Alt Med.

[38] Lee KB, Cho E, Kang YS (2014). Changes in 5-hydroxytryptamine and cortisol plasma levels in menopausal women after inhalation of clary sage oil. Phytother Res.

[39] Park H, Lim E, Zhao RJ, Oh SR, Jung JW, Ahn E, Lee ES, Koo S, Kim HY, Chang S, Shim HS, Kim KJ, Gwak YS, Yang CH (2015). Effect of the fragrance inhalation of essential oil from *Asarum heterotroipodes* on depression-like behaviors in mice. BMC Complement Alt Med.

[40] Satou T, Takahashi M, Kasuya H, Murakami S, Hayashi S, Sadamoto K, Koike K (2013). Organ accumulation in mice after inhalation of single or mixed essential oil compounds. Phytother Res.

[41] Satou T, Kasuya H, Takahashi M, Murakami S, Hayashi S, Sadamoto K, Koike K (2011). Relationship between duration of exposure and anxiolytic-like effects of essential oil from *Alpinia zerumbet*. Flavour Frag J.

[42] Vaudry D, Stork PJ, Lazarovici P, Elden LE. Signaling pathways for PC12 cell differentiation: making the right connections. Science. 2002;296:1648–9. 10.1126/science.1071552 12040181

[43] Okuda T, Haga T, Endou H, Ishihara T, Katsura I (2000). Identification and characterization of the high-affinity choline transporter. Nature Neurosci.

[44] WT S, Liao YF, TW W, Wang BJ, Shin YY (2012). Microgrooved patterns enhanced PC12 cell growth, orientation, neurite elongation, and neuritogenesis. J Biomed Mater Res.

[45] Denny JB (2006). Molecular mechanisms, biological actions, and Neuropharmacology of the growth-associated protein GAP-43. Curr Neuropharmacol.

